# Reduced Brain Gray Matter Volume in Patients With First-Episode Major Depressive Disorder: A Quantitative Meta-Analysis

**DOI:** 10.3389/fpsyt.2021.671348

**Published:** 2021-07-01

**Authors:** Ruiping Zheng, Yong Zhang, Zhengui Yang, Shaoqiang Han, Jingliang Cheng

**Affiliations:** Department of Magnetic Resonance Imaging, the First Affiliated Hospital of Zhengzhou University, Zhengzhou, China

**Keywords:** major depressive disorder, first-episode, voxel-based morphometry, signed differential mapping, meta-analysis

## Abstract

**Background:** The findings of many neuroimaging studies in patients with first-episode major depressive disorder (MDD), and even those of previous meta-analysis, are divergent. To quantitatively integrate these studies, we performed a meta-analysis of gray matter volumes using voxel-based morphometry (VBM).

**Methods:** We performed a comprehensive literature search for relevant studies and traced the references up to May 1, 2021 to select the VBM studies between first-episode MDD and healthy controls (HC). A quantitative meta-analysis of VBM studies on first-episode MDD was performed using the Seed-based d Mapping with Permutation of Subject Images (SDM-PSI) method, which allows a familywise error rate (FWE) correction for multiple comparisons of the results. Meta-regression was used to explore the effects of demographics and clinical characteristics.

**Results:** Nineteen studies, with 22 datasets comprising 619 first-episode MDD and 707 HC, were included. The pooled and subgroup meta-analysis showed robust gray matter reductions in the left insula, the bilateral parahippocampal gyrus extending into the bilateral hippocampus, the right gyrus rectus extending into the right striatum, the right superior frontal gyrus (dorsolateral part), the left superior frontal gyrus (medial part) and the left superior parietal gyrus. Meta-regression analyses showed that higher HDRS scores were significantly more likely to present reduced gray matter volumes in the right amygdala, and the mean age of MDD patients in each study was negatively correlated with reduced gray matter in the left insula.

**Conclusions:** The present meta-analysis revealed that structural abnormalities in the fronto-striatal-limbic and fronto-parietal networks are essential characteristics in first-episode MDD patients, which may become a potential target for clinical intervention.

## Introduction

Major depressive disorder (MDD), the third leading cause of global disease burden and a leading cause of disability worldwide, is a prevalent mental disorder caused by many complex factors, such as trauma, stress, psychological, and even some social or genetic factors (1). The pathophysiology of MDD has not yet to be fully elucidated and no preventive treatments are currently available. Thus, the investigation of the etiology, therapy and relevant biomarkers of MDD is of high importance for society and current research.

As a non-invasive imaging technique, structural magnetic resonance (MR) imaging has shown great potential value in elucidating the neuropathogenesis of psychiatric disorders. Structural MR imaging studies, which are paradigm free, are potentially able to make comparisons containing fewer biases across studies (2). Voxel-based morphometry (VBM) is a whole-brain and automatic technique which has accuracy comparable with manual volumetry and overcomes the technical limitations of region-of-interest approaches (1, 3). Notably, VBM has become an established research method to detect neuromorphometric abnormalities in subjects with various psychiatric disorders, such as schizophrenia, bipolar disorder, and obsessive–compulsive disorder, including MDD (4–11).

Previous VBM studies have demonstrated alterations in gray matter volume in MDD patients, however, the results vary considerably across studies. For example, Serra-Blasco et al., Stratmann et al. and Lu et al. did not find any significant gray matter difference between MDD patients and HC, whereas Zhang et al. and Kong et al. have detected increased gray matter volume (12–16). While, most studies have identified decreased gray matter volume in a wide range of brain regions, including the pre-supplementary motor area, the parietal-temporal regions, the frontal cortex, the temporal cortex, the cingulate cortex, the insular cortex, the parahippocampal gyrus, the hippocampus, the cerebellum and the orbitofrontal cortex (12, 16–24). These inconsistencies mainly might be contributed to the heterogeneity of MDD patients, which came from clinical and demographic characteristics of patients, the used imaging protocols and devices, the sample sizes and the technical methods of data acquisition and analysis. Two of the most important interferences are medications and chronic episodes, which are resulting in difficulty explaining the core pathophysiology of this disease independently from the potentially confounding factors mentioned above. In addition, MDD patients with comorbidity (such as anxiety disorders) are among the most frequently occurring psychiatric conditions and commonly present occur together (25) and often undifferentiated in many studies (26). Thus, we speculate that the results may have been confounded when MDD patients with/without comorbidity were grouped together in previous meta-analysis studies (1, 27–29). Therefore, to better understand the whole brain primary morphometric changes in MDD patients, the above-mentioned limitations need to be overcome in research. For this aim, we conducted subgroup meta-analysis to evaluate the potentially confounding effect including medication and comorbidity in first-episode MDD patients. To our knowledge, this is the first meta-analysis of VBM studies in patients with MDD that examines whether comorbidity affect the gray matter volumes or not with the latest version of anisotropic effect size seed-based d mapping (ES-SDM).

The ES-SDM is a coordinate-based meta-analytic technique (10, 11), which has some advantages over the previous approaches. Firstly, it allows all the useful information from contributing studies to be used in the same map including both positive and negative differences, which can prevent a particular voxel from appearing to be significant in opposite directions. Secondly, SDM has some complementary analyses, such as jack-knife, subgroup and meta-regression analyses, which can be used to assess the robustness and heterogeneity of the results (30). Since the updated version of ES-SDM, namely, the Seed-based d Mapping with Permutation of Subject Images (SDM-PSI) allows a FWE correction for multiple comparisons of the results, we will conduct a quantitative analysis using the SDM-PSI method to reflect intrinsic brain structure of MDD.

## Methods

### Study Selection

On the basis of the Preferred Reporting Items for Systematic Reviews and Meta-Analyses (PRISMA) (see Supplementary Table 1) statement (31), we performed a systematic and comprehensive literature search in PubMed, Embase, Web of Science, and Ovid databases for relevant studies published up to May 1, 2021. The search strategy was: “VBM,” or “voxel-based,” or “morphometry” or “voxel-based morphometry” and “depression,” or “depressive disorder,” or “major depression,” or “major depressive disorder,” or “depressed.” We also checked the reference lists and review articles to identify studies that may have been missed in the original search. Two researchers (R.P.Z and Z.G.Y) independently searched the articles. A study was included if it was: (1) written in English and peer reviewed, (2) enrolled patients diagnosed with first-episode MDD and a matched HC group, (3) used VBM to analysis whole-brain gray matter volume changes, (4) clearly reported three coordinates (x, y, z) in a stereotactic space (Talairach or MNI). The exclusion criteria were as follows: (1) no first-episode MDD patients versus HC, (2) using ROI or seed voxel-based analysis, (3) missing important information on the results (e.g., coordinates of significant clusters [*P* < 0.05]) even after contacting the corresponding author. If there were some inconsistent opinions, two researchers reached a consensus results through the discussion, and then moved to the following steps.

### Quality Assessment

The quality of all enrolled articles was independently accessed using a 10-point checklist by two authors (R.P.Z and Z.G.Y) (29, 32). The checklist included diagnostic criteria applied, demographic and clinical characteristics, the sample size, the quality of the reported results, the methods of image acquisition, imaging technique (see Supplementary Table 2). Although the checklist was not designed as an assessment tool, it still provided some objective indicators of the rigor of each study. If the rating results were inconsistent, the two authors reached a unified quality score by discussion. The final quality scores are shown in Supplementary Table 1.

### Voxel-Wise Meta-Analysis

The coordinate-based meta-analysis was conducted by using SDM-PSI software version 6.21 (www.sdmproject.com/software/), which has been described in detail in some studies (33, 34) and the SDM-PSI reference manual (https://www.sdmproject.com/manual/), here we only described it briefly. Its standard procedures include: calculation of the maps of the lower and upper bounds of possible effect sizes for each study separately based on the peak information, full anisotropy = 1, isotropic full width half maximum = 20 mm, and voxel = 2 mm; the mean analysis: estimation of the map of most likely effect size and its standard error, conducting multiple imputations of the maps of effect size of the individual studies, meta-analysis of these maps using a standard random-effects model, and Rubin rules to pool the different meta-analyses resulting from the multiple imputations; FWE correction for multiple comparisons using common permutation tests (*p* < 0.05); and finally use of threshold-free cluster enhancement (TFCE) in the statistical thresholding (*p* < 0.05, voxel extent ≥10).

Moreover, we conducted jackknife sensitivity, heterogeneity and meta-regression analyses using SDM. Jackknife sensitivity analysis was conducted to assess the reproducibility of the results by the procedure of repeating the meta-analysis after discarding one study each time (30). Heterogeneity analysis was performed to explore unexplained inter-study variability of the results. Although *I*^2^ statistics were used widely to assess the heterogeneity (*I*^2^ < 50% indicates low heterogeneity) in previous meta-analysis, *I*^2^ has been questioned for accuracy and reliability by some researchers (35).Thus we combined tau^2^ and *I*^2^ to explore inter-study heterogeneity (30). Egger's tests were calculated to assess potential publication bias (*p* < 0.05 indicates obvious publication bias). Meta-regression analysis was conducted to explore the potential effects of clinical variables (*p* < 0.00005, uncorrected, voxels > 10 indicates statistical differences), such as gender rations, mean age, education duration, illness duration, and severity of depression symptoms, by means of simple linear regression. Additional, two subgroup meta-analyses were performed to eliminate the effect of medication and comorbidity. Firstly, we conducted a subgroup analysis of first-episode medication-naïve MDD patients (*n* = 477) to eliminate confounders such as illness duration and previous antidepressant treatment. Secondly, two subgroup analysis of first-episode MDD patients without comorbidity (*n* = 402) and with comorbidity (*n* = 149) were performed to control the confounding factors of comorbidity.

## Results

### Included Studies and Sample Characteristics

**Figure 1:**
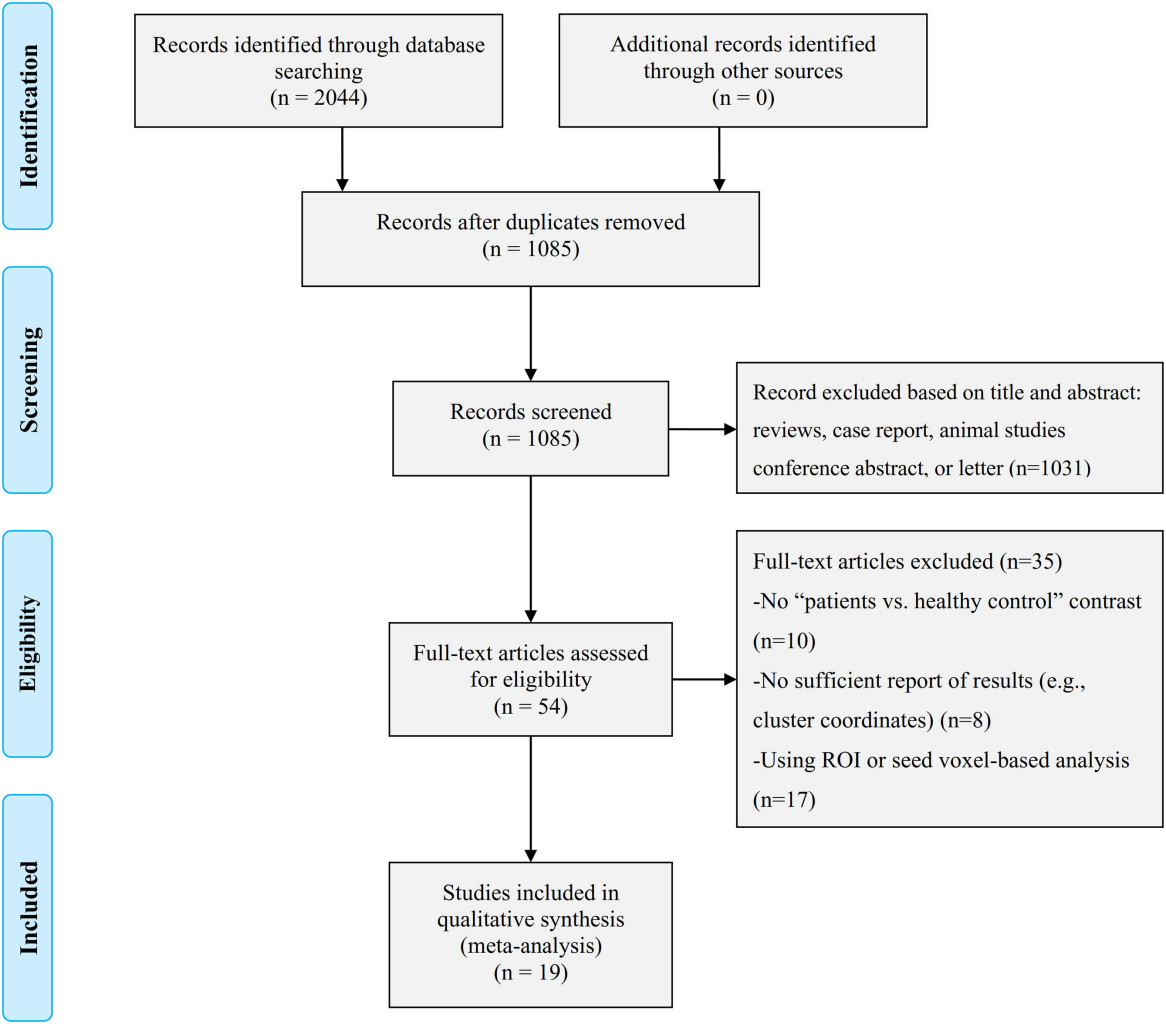
Search strategy used for the inclusion of the studies considered in the current meta-analysis.

The identification and attrition of the studies are shown in Figure 1. Finally, the search identified 2,044 studies, and only 19 studies met the inclusion criteria (7, 12–24, 36–40), of which three reported two separate experiments (7, 20, 39). Our final sample consisted of 619 first-episode MDD patients (371 females and 248 males; mean age 33.43 ± 4.71 years) and 707 HCs (403 females and 304 males; mean age 32.90 ± 4.79 years). In addition, some more basic information about the subjects is shown in Table 1. There was no significant difference in age, sex, education duration between MDD patients and HCs (all *p* > 0.05). Sixteen out of 22 datasets included patients who were medication-naïve, which included 477 medication-naïve MDD (288 females and 189 males; mean age 32.02 ± 4.21 years) patients and 533 HCs (307 females and 226 males; mean age 31.52 ± 4.16 years). Thirteen out of 22 datasets included patients who were without comorbidities, which included 372 patients with first-episode MDD (213 females and 159 males; mean age 36.54 ± 4.69 years) and 431 HCs (231 females and 200 males; mean age 36.41 ± 4.32 years).

**Table 1:** Demographic and clinical characteristics of subjects in the 22 voxel-based morphometry datasets included in the meta-analysis^a^.

**Study**	**Subjects (females**, ***n*****)**		**Age (Y)**		**Education (Y)**		**Illness duration (M)**	**Severity (scale type)**	**Comorbidity of other psychiatric disorder**	**Drug status**
	**FED**	**HC**	**FED**	**HC**	**FED**	**HC**				
Zhang et al. (40)	30 (21)	63 (39)	25.0	23.0	12.5	16.0	NA	26.5 (HAMD)	Anxiety disorders	Drug naïve
Liu et al. (7)	24 (15)	30 (14)	34.79	33.43	12.75	13.97	NA	25.5 (HAMD)	Negative	Drug status
Liu et al. (7)	21 (11)	30 (14)	34.14	33.43	13.29	13.97	NA	24.4 (HAMD)	Negative	Drug status
Yang et al. (39)	41 (30)	16 (12)	31.7	32.9	11.5	14.3	9.8	24.7 (HAMD)	May anxiety disorders	Drug naïve
Yang et al. (39)	43 (31)	68 (49)	30.1	29.8	11.9	16.0	8.2	23 (HAMD)	May anxiety disorders	Drug naïve
Igata et al. (38)	27 (12)	47 (12)	45.8	41.2	NA	NA	NA	21.8 (HAMD)	Negative	Drug naïve
Lu et al. (13)	30 (15)	26 (13)	34 31.42		15.06	16.69	NA	23.73 (HDRS)	Negative	Drug naïve
Chen et al. (36)	27 (14)	28 (14)	33	33	NA	NA	79	22 (HDRS)	Negative	Drug naïve
Kong et al. (12)	28 (17)	28 (14)	34.42	32.07	11.79	12.36	2.11	21.64 (HDRS)	Negative	Drug native
Lai et al. (19)	38 (20)	27 (15)	36.57	38.29	15.68	15.92	4.68	22.26 (HDRS)	Negative	Drug native
Stratmann et al. (15)	35 (21)	35 (19)	34.86	35.14	NA	NA	14.66	19.46 (HDRS)	Eleven patients had anxiety disorders	Thirty-three patients were on medication
Guo et al. (37)	24 (11)	44 (24)	NA	29.39	NA	NA	NA	NA	Negative	Drug naïve
Serra-Blasco et al. (14)	22 (15)	32 (23)	44	46	NA	NA	5.6	16 (HDRS)	Negative	All patients were on medication at the beginning of the study
Ma et al. (20)	17 (7)	17 (7)	26.71	24.24	12.35	13.82	2.59	25.58 (HDRS)	NA	Drug native
Ma et al. (20)	18 (7)	17 (7)	27.39	24.24	13.56	13.82	35.5	23.89 (HAMD)	NA	At least two classes of antidepressants
Wang et al. (23)	18 (9)	18 (9)	34	35	13	14	5	25 (HDRS)	Negative	Drug naive
Zhang et al. (16)	33 (16)	32 (15)	20.52	21.03	13.85	14.00	NA	37.67 (CES-D)	NA	Drug naive
Peng et al. (21)	22 (14)	30 (19)	46.7	45.9	11.2	12.5	8.6	18.5 (HDRS)	Negative	Five patients were taking antidepressants at the time of enrollment
Cheng et al. (17)	68 (47)	68 (47)	29.91	30.54	12.78	13.53	10.98	22.32 (HDRS)	Negative	Drug native
Lai et al. (18)	16 (11)	15 (11)	37.91	34.30	NA	NA	4.08	35.91 (HDRS)	PD	Drug native
Zou et al. (24)	23 (13)	23 (13)	31.1	36.6	11.9	12.4	7.6	24.4 (HDRS)	Negative	Drug naïve
Tang et al. (22)	14 (14)	13 (13)	29.5	29.46	11.43	12.23	5.22	NA	Four patients had a current comorbid diagnosis of GAD	Drug naive

a *Y = years; M = months; CES-D = Center for Epidemiological Studies Depression Scale; FED = first episode depression; GAD = general anxiety disorder; HC = healthy control; HDRS = Hamilton Depression Rating Scale; HAMD = Hamilton Depression Scale; NA = not available; PD = panic disorder*.

### Pooled Meta-Analysis

**Figure 2:**
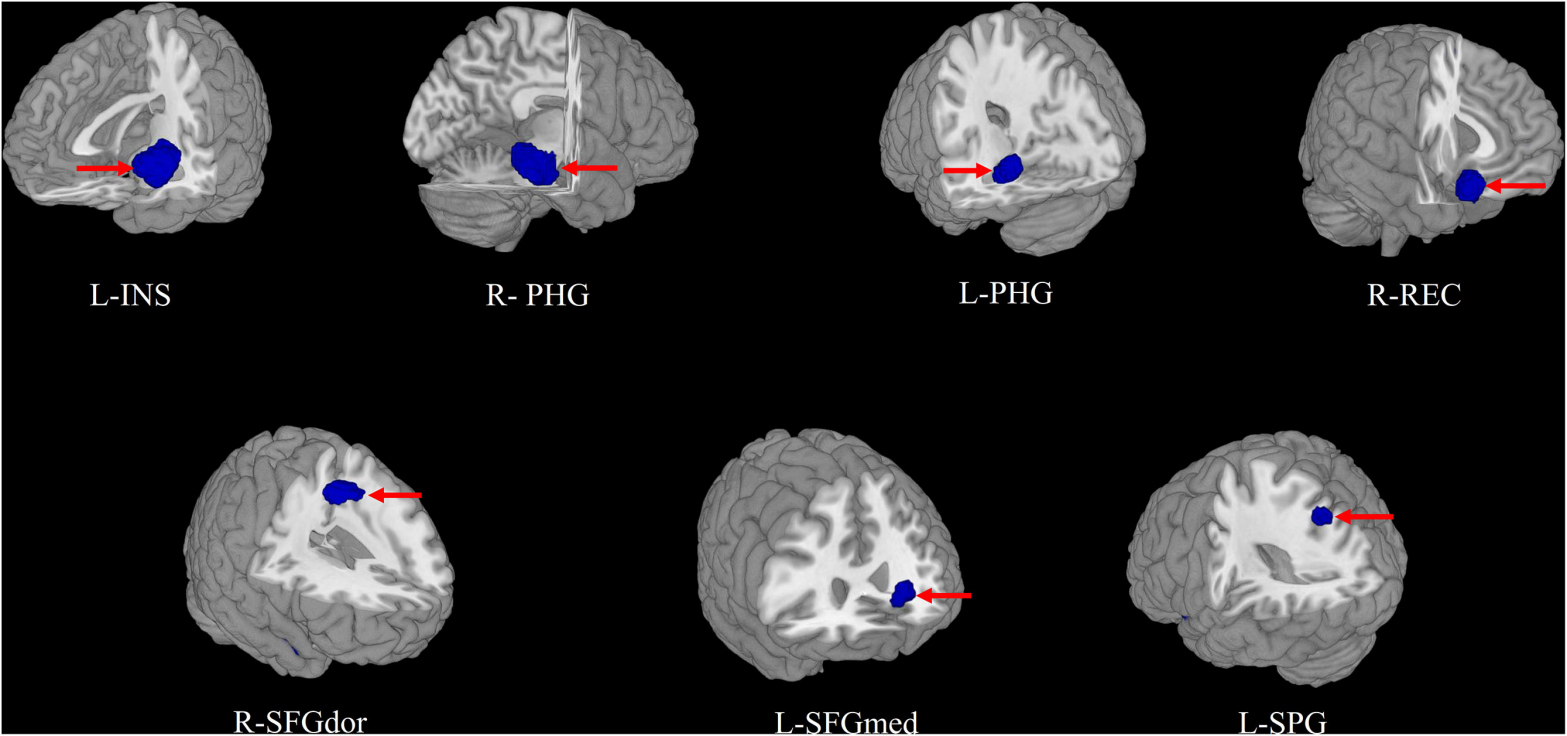
The areas of decreased (blue) gray matter volumes in patients with first episode depression (FED) compared with healthy controls in the pooled meta-analysis. INS, insula; PHG, parahippocampal gyrus; REC, gyrus rectus; SFGdor, superior frontal gyrus, dorsolateral; SFGmed, superior frontal gyrus, medial; SPG, superior parietal gyrus; L, left; R, right.

In the pooled meta-analysis, using a threshold of FWE-corrected *p* < 0.05, first-episode MDD patients showed gray matter volume reduction in the left insula (INS), bilateral parahippocampal gyrus (PHG) extending into the bilateral hippocampus (HIP), right gyrus rectus (REC), right dorsolateral part of superior frontal gyrus (SFGdor), right striatum, left medial part of superior frontal gyrus (SFGmed), and left superior parietal gyrus (SPG), compared with HCs. No increased gray matter volume was found in MDD patients (Figure 2 and Table 2).

**Table 2:** Regional differences in gray matter volume between FED patients and healthy controls in the pooled meta-analysis.

**Brain regions**	**MNI coordinates**			**SDM value**	***p*-value**	**Cluster**		**Jackknife sensitivity analysis**
	***x***	***y***	***z***			**No. of voxels**	**Cluster breakdown (no. of voxels)**	
***FED<HC***								
Left insula, BA 48	−44	2	−4	−2.062	0.000020623	1,477	Left insula, BA 38,45,47,48 (572) Left rolandic operculum, BA 48 (137) Left superior temporal gyrus, BA 21,22,38,48 (197) Left inferior frontal gyrus, opercular part, BA 6,44,48 (100) Left inferior frontal gyrus, triangular part, BA45,47,48 (58) Left inferior frontal gyrus, orbital part, BA38,47 (47) Left temporal pole, superior temporal gyrus, BA 21, 38, 48 (195)	20/21
Right parahippocampal gyrus, BA 30	24	−30	−14	−1.956	0.000092924	690	Right lingual gyrus, BA 27,30 (35) Right parahippocampal gyrus, BA 20,27,30,35,36,37 (126) Right hippocampus, BA20,30,35 (28) Right fusiform gyrus, BA 20,30 (48) Right cerebellum, hemispheric lobule III, BA 30 (29) Right cerebellum, hemispheric lobule IV/V, BA 30 (24)	20/21
Left parahippocampal gyrus, BA37	−28	−36	−12	−2.025	0.000046432	512	Left parahippocampal gyrus, BA 20,30,37 (119) Left hippocampus, BA 20,30,37 (63) Left fusiform gyrus, BA30,37 (84) Left lingual gyrus, BA30,37 (15)	20/21
Right gyrus rectus, BA 11	8	22	−18	−1.968	0.000077426	383	Right gyrus rectus, BA 11,25 (135) Right striatum (47) Right superior frontal gyrus, orbital part, BA 11,25 (21)	19/21
Right superior frontal gyrus, dorsolateral, BA 6	18	2	60	−1.745	0.000381887	145	Right superior frontal gyrus, dorsolateral, BA 6,8 (69) Right supplementary motor area, BA 6 (25)	19/21
Left superior frontal gyrus, medial, BA10	−8	50	6	−1.604	0.001068294	95	Left superior frontal gyrus medial, BA10,32 (48) Left anterior cingulate / paracingulate gyri, BA10,32 (22)	19/21
Left superior parietal gyrus, BA7	−30	−70	56	−1.603	0.001068294	55	Left superior parietal gyrus, BA7 (54)	19/21

*FED, first-episode depression; HC, healthy control; MNI, Montreal Neurological Institute; SDM, signed differential mapping; BA, Brodmann area; FEW-corrected p < 0.05*.

### Reliability Analyses

The results of whole-brain jackknife sensitivity analysis between first-episode MDD and HC (Supplementary Table 3) showed that the gray matter volume reduction in the left INS and left PHG was highly replicable, as this finding was replicated across all 21 combinations of the datasets. The gray matter volume reduction in the right PHG was also significant in all but one of the datasets. The gray matter volume reduction in the right GR, the right SFGdor, the left SFGmed, the left SPG was also significant in all but two of the datasets.

### Subgroup Analysis of First-Episode, Medication-Naïve MDD (16 Datasets)

The subgroup analysis of first-episode, medication-naïve MDD included 16 datasets comprising 477 medication-naïve MDD patients and 533 HCs. This analysis revealed that MDD patients, relative to controls, showed decreased gray matter volume in the left INS, right REC, right SFGdor, left SFGmed, left SPG, and right amygdala.

### Subgroup Analysis of First-Episode MDD Without Comorbidities (13 Datasets)

The subgroup analysis of first-episode MDD without comorbidities included 13 datasets comprising 372 patients with first-episode MDD and 431 HCs. This analysis revealed gray matter volume decreases in the left INS, bilateral PHG, and right SFGdor in first-episode MDD patients without comorbidities compared with HCs (Supplementary Table 4).

### Subgroup Analysis of First-Episode MDD With Comorbidities (6 Datasets)

The subgroup analysis of first-episode MDD with comorbidities included 6 datasets comprising 179 patients with first-episode MDD and 210 HCs. This analysis showed gray matter volume decreases in the right SFGorb, left SFGmed, left SPG in first-episode MDD patients with comorbidities compared with HCs (Supplementary Table 4).

### Analysis of Heterogeneity and Publication Bias

Heterogeneity analyses exhibited low inter-study variability in the left INS. Moreover, we used a funnel plot and Egger's test to assess potential publication biases for the brain regions identified in the meta-analysis. In all seven clusters, the funnel plots were found to be roughly symmetric, except for only one region (left PHG, Egger test: *P* = 0.027), and Egger's tests did not detect significant differences, suggesting that there was nearly no publication bias in our main findings (Supplementary Figure 1).

### Meta-Regression Analyses

**Figure 3:**
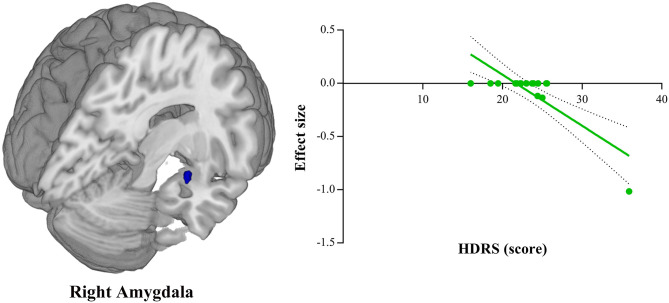
Meta-regression analysis shows that Hamilton Depression Rating Scale (HDRS) score of depressed patients is negatively correlated with gray matter volume in the right amygdala. In the graphs, the effect sizes needed to create this plot have been extracted from the peak of maximum slope significance, and each study is represented as a dot. The regression line (meta-regression signed differential mapping slope) is shown.

**Figure 4:**
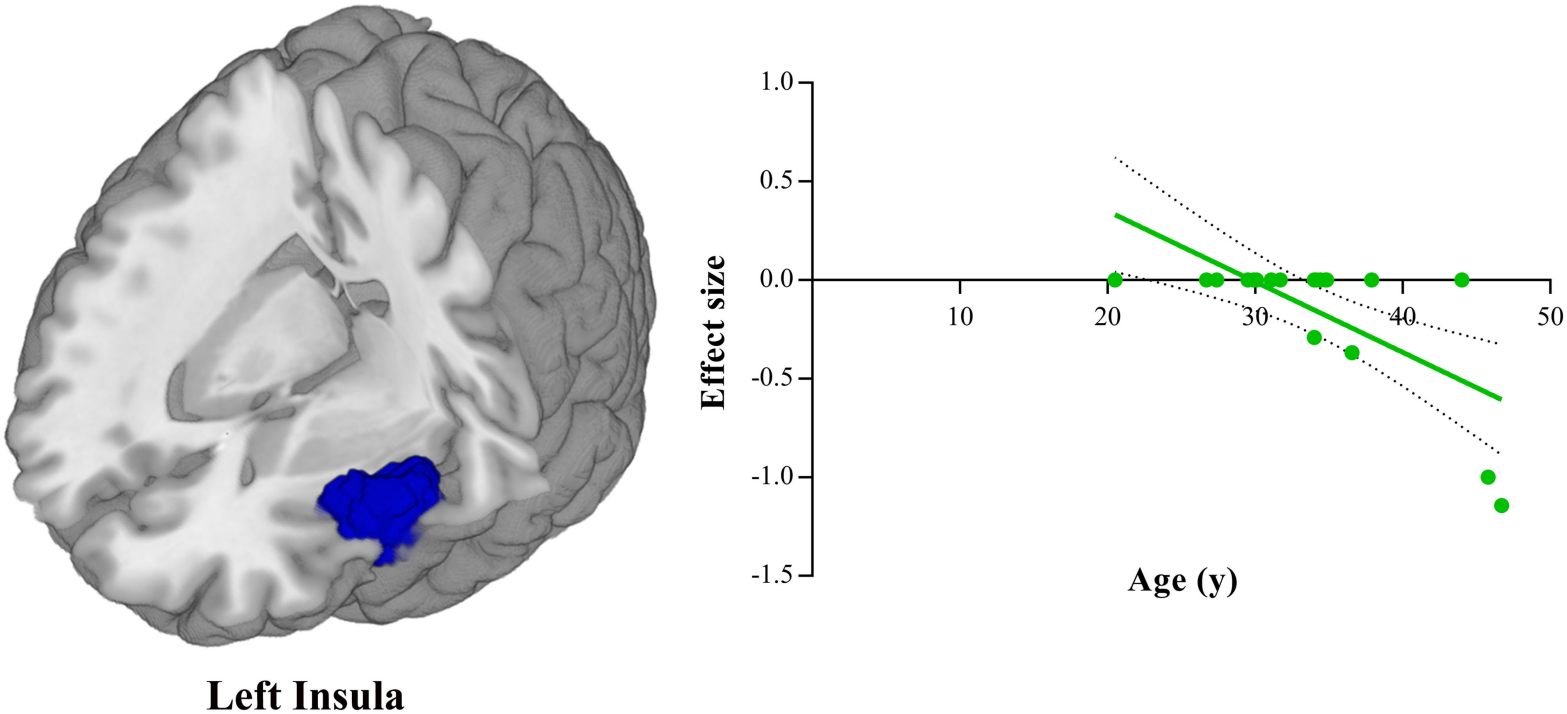
Meta-regression analysis shows that mean age of depressed patients is negatively correlated with gray matter volume in the left insula. In the graphs, the effect sizes needed to create this plot have been extracted from the peak of maximum slope significance, and each study is represented as a dot. The regression line (meta-regression signed differential mapping slope) is shown.

Meta-regression analyses showed that the HDRS score was negatively correlated with the gray matter volume in the right amygdala (BA 34; MNI coordinate: x = 28, y = 0, z = −20; SDM-Z = −1.906, *P* = 0.0001, voxel = 16) (Figure 3). The gray matter volume in the left insula (BA 48; MNI coordinate: x = −44, y = 10, z = −8; SDM-Z = −3.605, *P* = ~0, voxel = 1,055) was shown to be modulated by age (Figure 4). However, this result should be interpreted with caution as it was driven by only three studies. No linear associations with sex ratio, education duration and illness duration were observed.

## Discussion

VBM is the most common way to identify abnormal gray matter volume in the clinical diseases. Our study integrated the findings from 19 VBM studies using AES-SDM, in which first-episode MDD patients were compared with HC. Without the influence of recurrence and external tasks, this whole-brain meta-analysis, comparing gray matter volume between first-episode MDD patients and HC, could reflect the intrinsic relationship between structural changes and pathologic processes in MDD. Brain regions with increased gray matter volume were not observed. In addition, to clarify the pathological mechanisms of pure MDD patients without comorbidity, we further conduct a subgroup. Both pooled and subgroup meta-analysis identified decreased gray matter volume in the fronto-striatal-limbic circuits. And, this meta-analysis founded that the HDRS score was negatively correlated with the gray matter volume in the right amygdala and the age was negatively correlated with gray matter volume in the left insula.

Most of the brain regions with reduced gray matter volume were located in the limbic system, including the left INS, bilateral PHG, bilateral HIP. The limbic system has been theoretically identified to play an important role in the pathophysiology of MDD by regulating the balance between experience, emotion and behavior, motivation and long-term memory (41, 42), because it has widespread connections to extensive cortical areas known as the neuroanatomical circuits of mood regulation (43). As an important part of limbic system, the INS has extensive connectivity with fronto-limbic regions, which may partly explain the difficulties in cognitive and emotional integration that characterize the clinical manifestations of MDD (12, 15, 19, 38, 44–50). Although many neuroimaging studies, involving in VBM and fMRI, have demonstrated the INS was concerning with MDD patients, the findings were inconsistent. Peng et al. showed a volume reduction of the bilateral insular in first-episode MDD patients (28). Kong et al. founded gray matter volume of the right insular increased in medication-naïve MDD patients (12), while a reduction of gray matter volume in the left insular was founded in first-episode medication-naïve MDD patients in a study conducted by Lai and Wu (19). Our findings are supported by many previous VBM studies that demonstrated a volume reduction of the left insular lobe in MDD patients (19, 51). This finding may reflect the heterogeneity of the right insular in MDD. It is also likely reflecting differential responses to the confining environment, such as the MRI scanner, and the different characteristics of subjects. Further studies are needed to determine their relative contributions to MDD pathology. The HIP and PHG, involved in the pathophysiology of MDD, are also important components of limbic system and limbic-cortical-striatal-pallidal-thalamic networks (52). They are assigned a pivotal role in assessing novel items, information retrieval success, visual memory, spatial memory, and recollection memory (53–55), and therefore damages to these regions could lead to diverse symptoms in MDD. Numerous neuroimaging studies have consistently demonstrated the deficits in gray matter volume in the HIP or PHG in various MDD samples, involved in first-episode patients, recurrent patients, medicated patients and medication-naïve patients, suggesting that the altered volume of HIP or PHG might be a trait-related biomarker to characterize MDD (7, 15, 56–60). However, results from these study differ in localization of HIP or PHG volume reduction with studies showing bilateral (7, 17, 24, 61, 62), left unilateral (21, 63, 64), and right unilateral (15, 18, 49) atrophy, which might be attributed to some mixed factors, such as sex, age, recurrences, medication, illness severity, educational level, and the magnetic field strength of MR scanner. In the present study, we detected the decreased gray matter volume in the bilateral HIP and HPG in the first-episode patients, consistent with other studies (7, 17, 24). An explanation for HIP atrophy in MDD is provided by the vulnerability hypothesis, suggesting that HIP atrophy is a pre-existing risk factor for MDD (65), and is therefore already evident in first-episode patients. In conclusion, our results of the present study suggest that the reduced gray matter volume of left INS, bilateral HIP and HPG could serve as neuroimaging biomarker for diagnosing MDD.

The striatum (composed of the putamen, the caudate, and the ventral striatum) is an important part of basal ganglia (60, 66). The striatum is involved in the fronto-striatal-limbic circuitry, and it plays an important role in motor and cognitive control, social learning and reward processing (50). Converging evidence suggests that the gray matter volume of striatum was decreased in the initial presentation of MDD patients (67–69), although not all studies replicated this finding. In the present study, we detected a decrease in the right striatum. The role of the striatum in MDD is not only supported by VBM studies but also by rs-fMRI studies. Lai et al. showed that gray matter volume decreased in the bilateral striatum, and this study was included in the present meta-analysis (18). However, we observed the decreased gray matter volume in the striatum on the right side. It is noteworthy that their study included small sample size (16 MDD patients, 15 healthy controls), which may lead to inaccurate results. In addition, rs-fMRI has shown that striatal activity was reduced in reward system defects (60, 70), and decreased reward network connections were found to be associated with depression severity (71). Meanwhile, functional studies have also confirmed the presence of metabolic abnormalities in MDD in the striatum (71). A minority of results does not same in localization of striatum volume loss with studies showing bilateral and right unilateral atrophy, which might be due to potential influencing factors like sex. As the Dluzen et al. found, estrogen appears to have a neuroprotective effect on the striatum, implicating decreased vulnerability in this region in females and increased vulnerability in males (72). Taken together, these findings suggest that the altered volume of striatum may contribute to internal pathophysiology in MDD.

We also found decreased gray matter volume in the right SFGdor and left SFGmed in first-episode MDD patients compared with controls. The dorsolateral prefrontal cortex (DLPFC) and medial prefrontal cortex (MPFC) are important components of the prefrontal lobe (73–75). These findings were in accordance with two postmortem studies on depressive patients which found decreased neuronal and glial cells respect to density, number and size in the prefrontal cortex (76, 77). According to previous studies (60, 78), the prefrontal regions have been considered to be the most common regions to manifest anatomic abnormalities in MDD. The DLPFC is critical components of frontal lobe, and plays an essential role in emotional, motivational, attentional, and executive functions (60, 78). Some studies have proved that the reduced volume of DLPFC correlates well with the hypoactivation during working memory updating and during conscious negative emotion processing in fMRI studies (79–81). The MPFC is a crucial cortical region that integrates information from numerous cortical and subcortical areas and converges updated information to output structures. It has been implicated in a variety of social, cognitive, and affective functions that are commonly impaired in mental illness (82). In recent years, MPFC has aroused increasing attention for its role in depression (83). Previous studies have showed that abnormal functional activity of MPFC has related to altered self-reflection and rumination and MPFC has also been implicated in emotion-regulation process, particularly in the down-regulation of negative affect (84). Our findings of significant gray matter loss in the MPFC replicate previous data (68, 85). Some researchers even supposed that highly variable MPFC-to-DLPFC connectivity may signify weaknesses in brain circuits responsible for cognitive control, and could be related to depressive deficits in executive functioning such as difficulty inhibiting emotional distraction (86, 87). In line with these results, we may speculate that the atrophy of DLPFC and MPFC are some reasons of the occurrence of MDD, and could serve as a neuroimaging biomarker for diagnosing MDD.

We observed decreased gray matter volume in first-episode MDD patients in the left SPG compared with HC. The SPG is part of the default-mode network, and it is involved in the organization, decision making, emotional processing, cognitive changes and predictions of rewards and so on (88, 89). The SPG is also involved in fronto-parietal network. Lai et al. showed decreased gray matter volume has been noted in fronto-parietal regions in MDD patients compared to HC, and then the gray matter volume of fronto-parietal regions increased after medication treatment (90). Cole et al. showed fronto-parietal network (along with other cognitive control networks) plays an important role in against mental disease via its widespread functional connectivity with other networks (91). Alterations in fronto-parietal network functional connectivity have been identified in a number of mental disorders, including depression (92). Some researchers have proved that inefficiency of the fronto-parietal circuit results in lower cognitive control, and then leads to problems with flexible cognition and executive functions, and could be the cause of more typical symptoms of depression like persistent rumination which are very often present in depressive disorders (93). Together with our findings, this suggests that fronto-parietal circuit serves as an important role in cognitive control networks, and alteration of gray matter volume of fronto-parietal regions may lead severe mental disease, including MDD.

To clarify the pathological mechanisms of pure MDD patients without comorbidity, we further conduct a subgroup analysis. Both pooled and subgroup meta-analysis identified decreased gray matter volume in the fronto-striatal-limbic circuits. As we all know, MDD and some other psychiatric disorders, such as anxiety disorder, panic disorder and so on, have an overlap of clinical symptoms, which suggests that they may share similar neurological mechanisms (50, 94, 95). And, patients with MDD often have considerable comorbidity, such as anxiety (96, 97). Then we speculated the results may have been confounded when MDD patients with comorbidity were grouped together in the previous studies. However, despite the clinical importance of comorbidity in MDD, few neuroimaging studies have focused on its brain structural alterations, or its difference from pure depression and depression with comorbidity (98). Peng et al. found that anxious depression had smaller gray matter volume in the fronto-limbic circuits (right inferior frontal gyrus and orbital frontal gyrus), which were in line with our results partly, relative to both non-anxious depression and healthy controls. The part difference may result from that the MDD patients with comorbidity in our meta-analysis include not just anxiety, but other comorbidity, such as panic disorder. Above all, although it is difficult to determine the factor contributing most to the smaller gray matter volumes, some articles have showed that the presence of comorbidity did not affect the rate of response to pharmacotherapy for depression (99), which to some extent was in line with our results that MDD with or without comorbidity may share the similar neurological mechanisms.

### Limitations

Some limitations of the current meta-analysis are well-acknowledged. Firstly, the sample size was not very large, especially in the subgroup analysis and meta-regressions, although they were more numerous than in many previous studies (26–29, 100). Secondly, despite controlling for the age and gender, the included studies in the present study varied in terms of the data acquisition, analysis techniques, demographic and clinical characteristics, which can lead to the impact of such heterogeneity on our meta-analysis. Thirdly, the accuracy of our voxel-wise meta-analysis may have been limited, because the accuracy was not derived from an original study rooted in raw statistical image but instead from published studies. Fourthly, due to the limitations of the current researches, we can't ensure all patients enrolled in comorbidity group were MDD patients with comorbidity. In the future study, more independent efforts about comorbidity should be made. Finally, to better understand the core mechanism of MDD, more longitudinal investigations of first-episode medication-naïve patients should be conducted to determine the causal relationships between clinical features and neuroimaging findings.

## Conclusions

The results of meta-analysis of all VBM studies implicate regional gray matter reduction in first-episode MDD patients is neural networks of fronto-striatal-limbic and fronto-parietal involved in the emotional and cognitive processing. When the confounding influence of medication or comorbidity was excluded, the subgroup meta-analysis results still focused on the fronto-striatal-limbic and fronto-parietal networks. The meta-regression analysis suggested that structural abnormality in the right amygdala may also be associated with the severity of depressive symptoms and structural abnormality in the left insula may be modulated by age. As such, our findings strongly implicate that the cause of MDD may be the abnormal gray matter volume of some certain brain areas, but whether the abnormal function connection of fronto-striatal-limbic and fronto-parietal networks is still unknown. To understand the complex pathogenic mechanism of MDD associated with fronto-striatal-limbic and fronto-parietal networks in the brain, further neuroimaging studies with a large number of subjects and sophisticated design should be pursued.

## Data Availability Statement

The original contributions presented in the study are included in the article/Supplementary Material, further inquiries can be directed to the corresponding author/s.

## Author Contributions

RZ and YZ conceived and designed the study. RZ and ZY are responsible for data acquisition. RZ and SH drafted the initial manuscript. JC, YZ, and SH reviewed and revised the manuscript. RZ and JC took responsibility for the paper. All authors read and approved the final manuscript.

## Conflict of Interest

The authors declare that the research was conducted in the absence of any commercial or financial relationships that could be construed as a potential conflict of interest.
