# Supplementary material for: Reduced Brain Gray Matter Volume in Patients With First-Episode Major Depressive Disorder: A Quantitative Meta-Analysis
Source: Front Psychiatry. 2021 Jul 1;12:671348. doi: 10.3389/fpsyt.2021.671348 (PMC8282212; doi:10.3389/fpsyt.2021.671348)
Supplement: Supplementary file 1 [file Data_Sheet_1.docx]

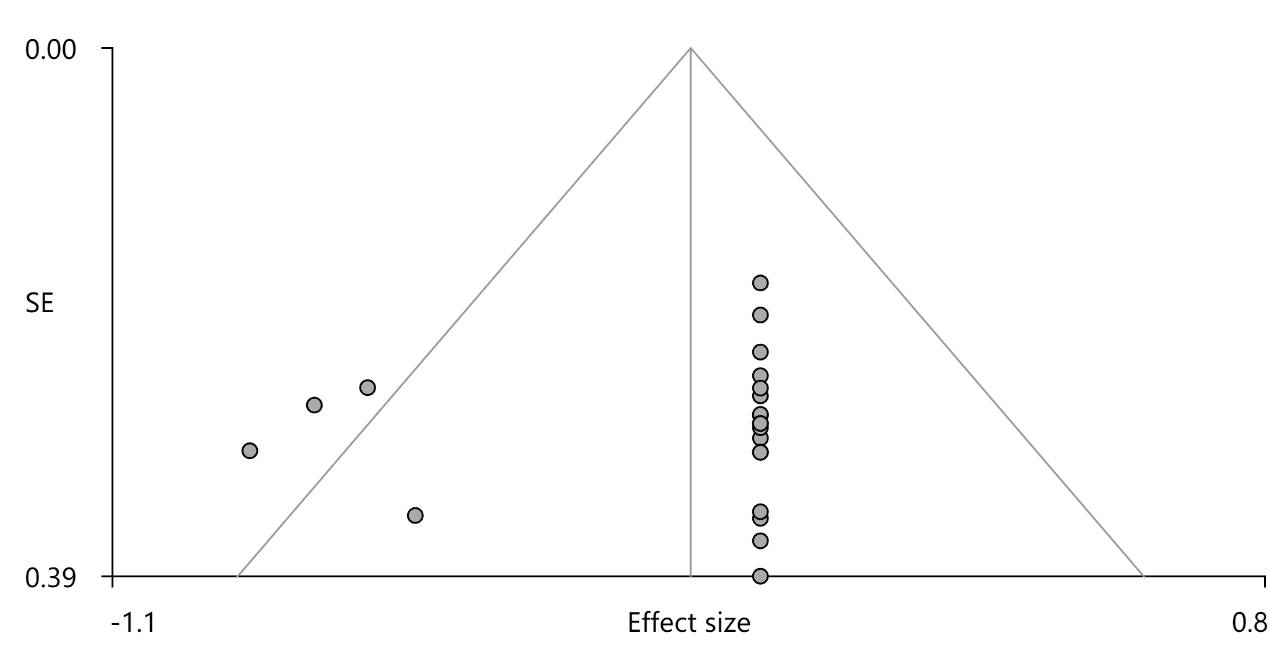


Figure S1.1 Funnel plot of left insula (Egger test: *p* = 0.502)


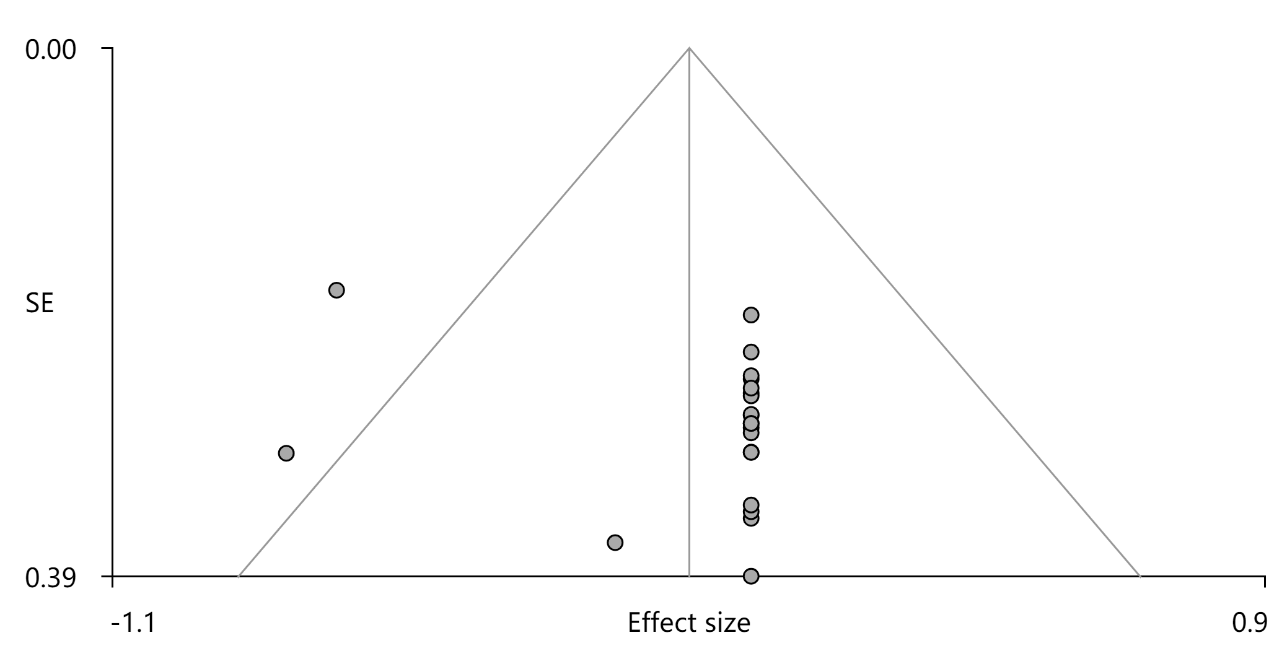


Figure S1.2 Funnel plot of right parahippocampal gyrus (Egger test: *p* = 0.121)


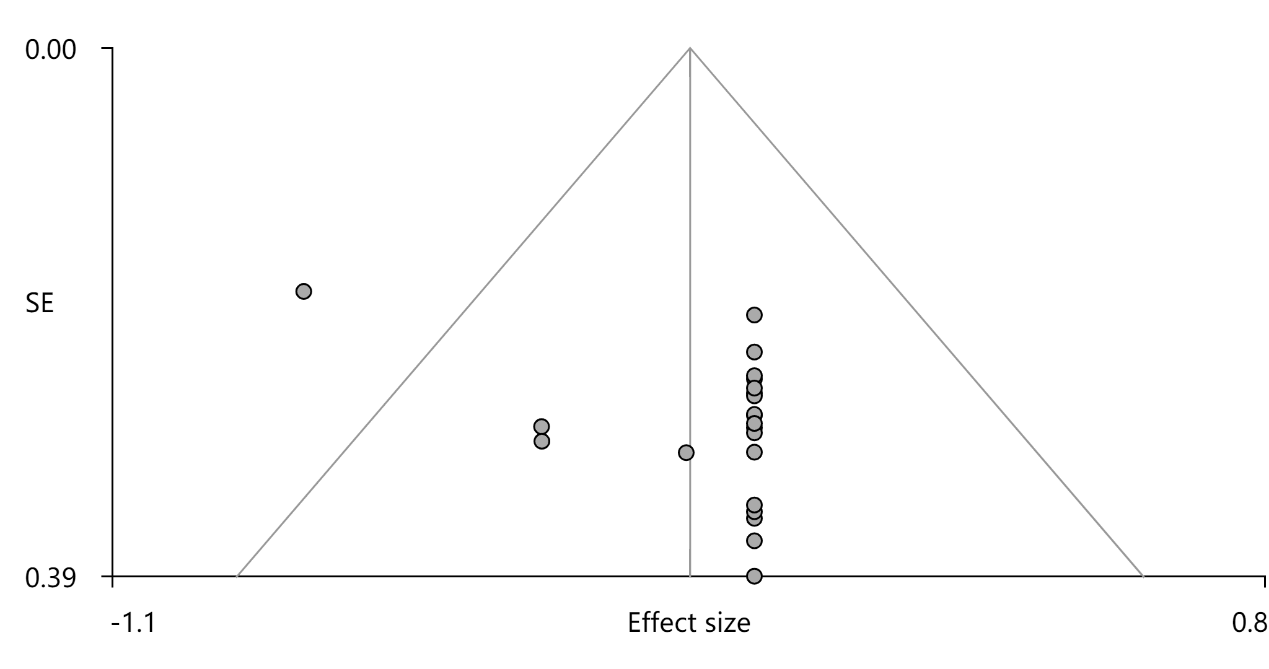


Figure S1.3 Funnel plot of left parahippocampal gyrus (Egger test: *p* = 0.031)


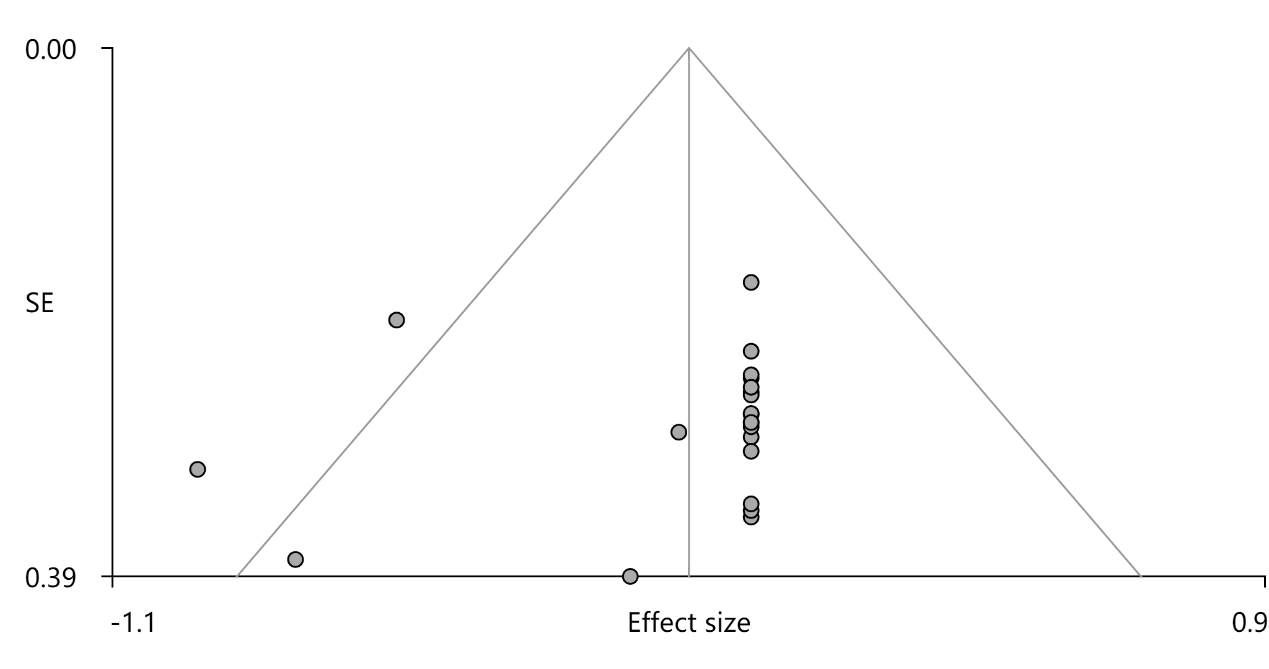


Figure S1.4 Funnel plot of right gyrus rectus (Egger test: *p* = 0.635)


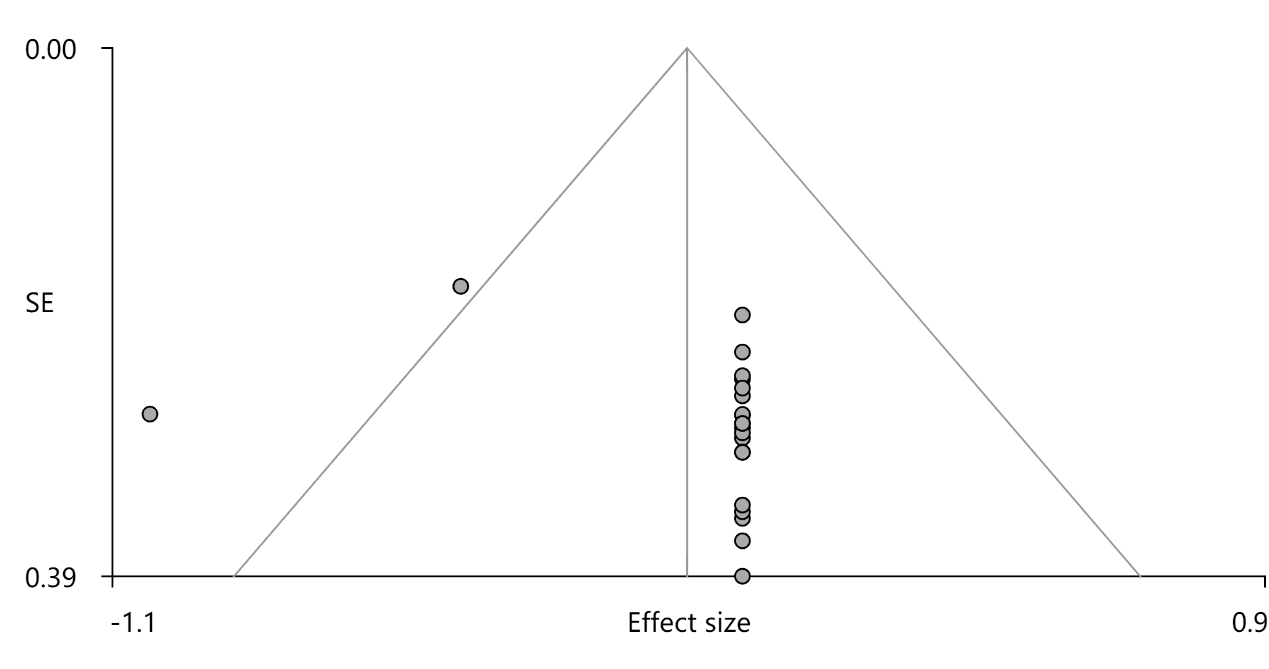


Figure S1.5 Funnel plot of right superior frontal gyrus, dorsolateral (Egger test: *p* = 0.153)


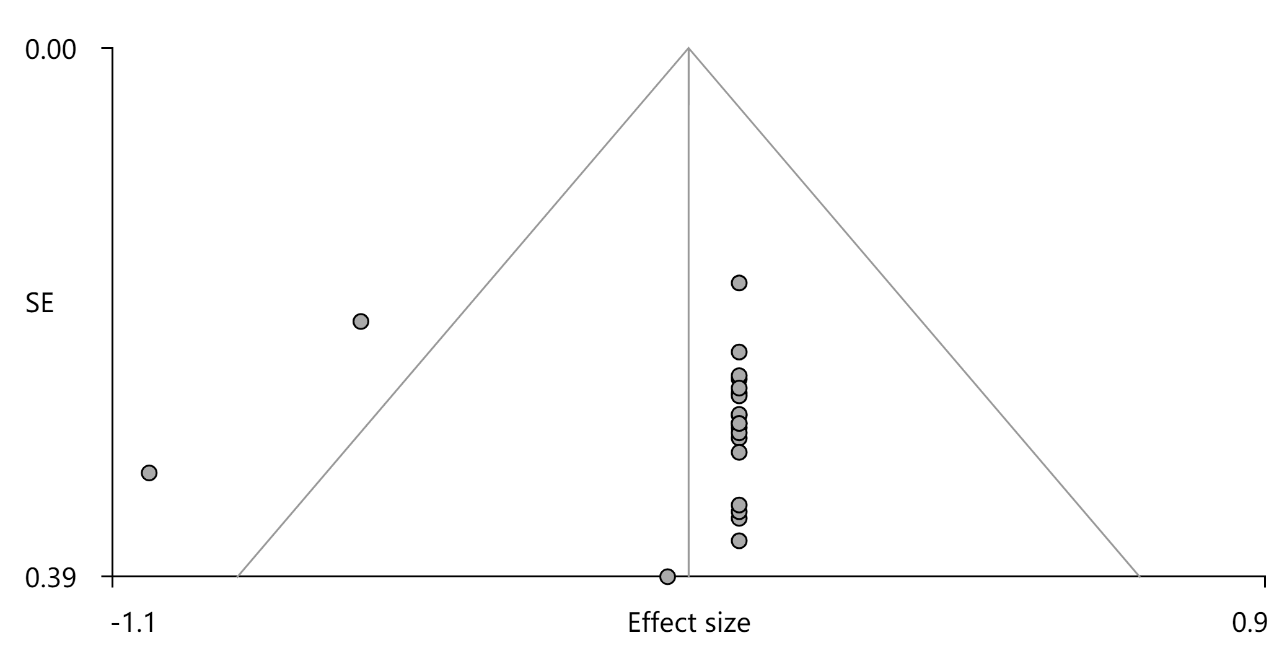


Figure S1.6 Funnel plot of left superior frontal gyrus, medial (Egger test: *p* = 0.722)


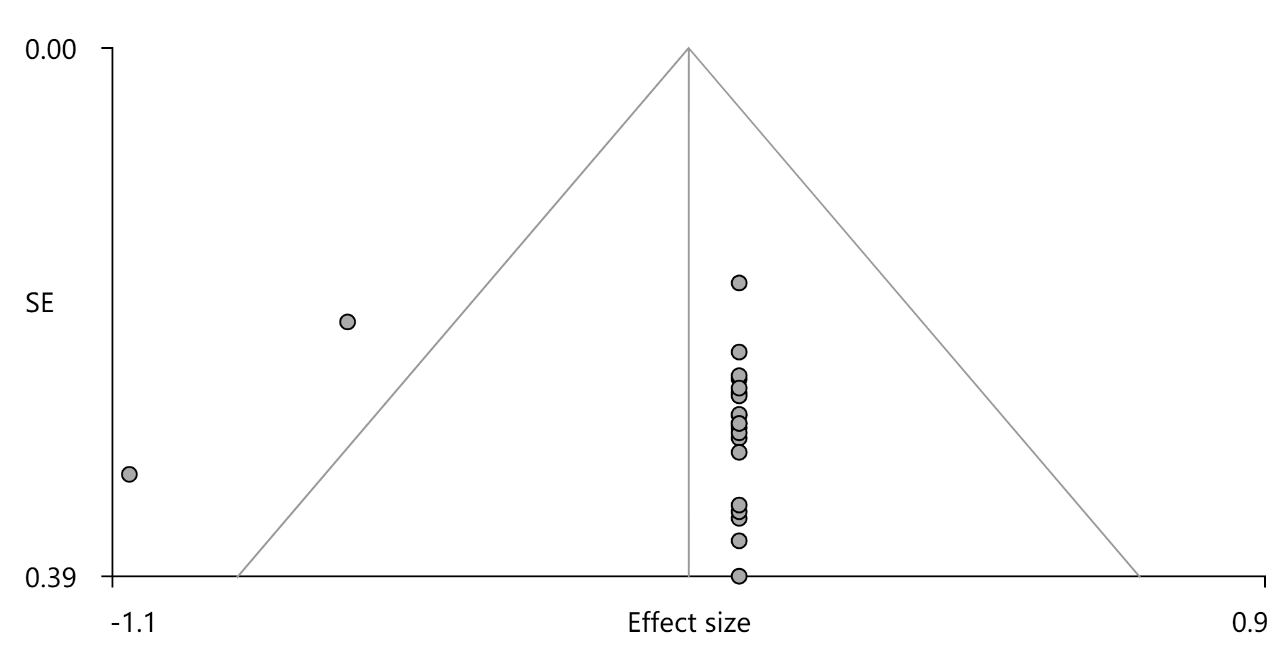


Figure S1.7 Funnel plot of left superior parietal gyrus (Egger test: *p* = 0.645)
