# Supplementary material for: Reduced Brain Gray Matter Volume in Patients With First-Episode Major Depressive Disorder: A Quantitative Meta-Analysis
Source: Front Psychiatry. 2021 Jul 1;12:671348. doi: 10.3389/fpsyt.2021.671348 (PMC8282212; doi:10.3389/fpsyt.2021.671348)
Supplement: Supplementary file 3 [file Table_2.docx]

**Table S2.** Quality Assessment Checklist (When criteria were partially met, 0.5 points were assigned)

| **Category 1: Participants** | Score (0/0.5/1) |
| --- | --- |
| 1. Patients were evaluated prospectively, specific diagnostic criteria were applied, and demographic data were reported.  2. Comparison participants were evaluated prospectively, psychiatric and medical illnesses were excluded.  3. Important variables (e.g., age, sex, illness duration, onset, medication status, BMI, comorbidity, intelligence quotient, i.e. IQ, handedness) were checked either by stratification or statistically.  4. Sample size per group > 10. | |
| **Category 2: Methods for image acquisition and analysis** | |
| 5. Whole brain analysis was automated with no a priori regional selection.  6. Magnet field strength > 1.5T.  7. MRI slice-thickness ≤ 3 mm and more than 1 slice was identified and traced.  8. Zero gap width.  9. Coordinates reported in a standard space.  10. The imaging technique used was clearly described so that it could be reproduced.  11. Measurements were clearly described so that they could be reproduced. | |
| **Category 3: Results and conclusions** | |
| 12. Statistical parameters for significant and important non-significant differences were provided.  13. Conclusions were consistent with the results obtained and the limitations were discussed. | |
| TOTAL /13 | |
