# Supplementary material for: Reduced Brain Gray Matter Volume in Patients With First-Episode Major Depressive Disorder: A Quantitative Meta-Analysis
Source: Front Psychiatry. 2021 Jul 1;12:671348. doi: 10.3389/fpsyt.2021.671348 (PMC8282212; doi:10.3389/fpsyt.2021.671348)
Supplement: Supplementary file 4 [file Table_3.docx]

**TABLE S3.** Jackknife sensitivity analyses of the studies comparing FED with HC included in the pooled meta-analysis

| **Discarded study** | **FED < HC** | | | | | | |
| --- | --- | --- | --- | --- | --- | --- | --- |
|  | L-INS | R-PHG | L-PHG | R-REC | R-SFGdor | L-SFGmed | L-SPG |
| Zhang et al. (2021) | N | Y | N | Y | N | N | N |
| Liu et al. (2019) | Y | Y | Y | Y | Y | Y | Y |
| Liu et al. (2019) | Y | Y | Y | Y | Y | Y | Y |
| Yang et al. (2017) | Y | Y | Y | N | Y | N | N |
| Yang et al. (2017) | Y | Y | Y | N | Y | N | N |
| Igata et al. (2017) | Y | Y | Y | Y | Y | Y | Y |
| Lu et al. (2016) | Y | Y | Y | Y | Y | Y | Y |
| Kong et al. (2014) | Y | Y | Y | Y | Y | Y | Y |
| Lai et al. (2014) | Y | Y | Y | Y | N | Y | Y |
| Stratmann et al. (2014) | Y | Y | Y | Y | Y | Y | Y |
| Guo et al. (2014) | Y | Y | Y | Y | Y | Y | Y |
| Serra et al. (2013) | Y | Y | Y | Y | Y | Y | Y |
| Ma et al. (2012) | Y | Y | Y | Y | Y | Y | Y |
| Ma et al. (2012) | Y | Y | Y | Y | Y | Y | Y |
| Wang et al. (2012) | Y | Y | Y | Y | Y | Y | Y |
| Zhang et al. (2012) | Y | Y | Y | Y | Y | Y | Y |
| Peng et al. (2011) | Y | Y | Y | Y | Y | Y | Y |
| Cheng et al. (2010) | Y | N | Y | Y | N | Y | Y |
| Lai et al. (2010) | Y | Y | Y | Y | Y | Y | Y |
| Zou et al. (2010) | Y | Y | Y | Y | Y | Y | Y |
| Tang et al. (2007) | Y | Y | Y | Y | Y | Y | Y |
| Total | 20/21 | 20/21 | 20/21 | 19/21 | 19/21 | 19/21 | 19/21^[[1]](#footnote-1)^ |

1. Abbreviations: FED = first-episode depression; HC = healthy control; INS = insula; REC = gyrus rectus; SFGdor = superior frontal gyrus, dorsolateral; PHG = parahippocampal gyrus; SFGmed = superior frontal gyrus, medial; SPG= superior parietal gyrus; R = right; L = Left; Y = yes; N = not [↑](#footnote-ref-1)
