# Supplementary material for: Reduced Brain Gray Matter Volume in Patients With First-Episode Major Depressive Disorder: A Quantitative Meta-Analysis
Source: Front Psychiatry. 2021 Jul 1;12:671348. doi: 10.3389/fpsyt.2021.671348 (PMC8282212; doi:10.3389/fpsyt.2021.671348)
Supplement: Supplementary file 5 [file Table_4.docx]

**TABLE S4. Regional differences in gray matter volume between FED patients and healthy controls in the subgroup meta-analysis**

| **Brain regions** |  | **MNI coordinates** | | |  | **SDM value** | ***p*-value** |  | **Cluster** | |  | **Jackknife sensitivity analysis** |
| --- | --- | --- | --- | --- | --- | --- | --- | --- | --- | --- | --- | --- |
|  |  | *x* | *y* | *z* |  |  |  |  | No. of voxels | Cluster breakdown (no. of voxels) |  |  |
| Subgroup meta-analysis of studies with medication- naïve | | | | | | | | | | | | |
| *FED < HC* | | | | | | | | | | | | |
| Left insula, BA 48 |  | -44 | 2 | -6 |  | -1.888 | 0.000108361 |  | 1539 | Left insula, BA 38, 47,48(642)  Left rolandic operculum, BA 48 (171)  Left superior temporal gyrus, BA 21, 38,48 (195)  Left inferior frontal gyrus, opercular part, BA 6,44,48 (99)  Left inferior frontal gyrus, triangular part, BA45,47,48 (31)  Left inferior frontal gyrus, orbital part，BA38,47(27)  Left temporal pole, superior temporal gyrus, BA 21, 38, 48 (149) |  | 16/16 |
| Right gyrus rectus, BA 11 |  | 4 | 22 | -26 |  | -1.945 | 0.000087738 |  | 685 | Right gyrus rectus, BA 11,25(176)  Right striatum (80)  Right superior frontal gyrus, orbital part, BA 11,25(41)  Right superior frontal gyrus, medial part, BA 11 (37)  Left gyrus rectus, BA 11,25(40)  Right olfactory cortex, BA 11,25(29)  Left olfactory cortex, BA 11,25(22)  Right anterior cingulate / paracingulate gyri, BA 11,25(6) |  | 16/16 |
| Right superior frontal gyrus, dorsolateral, BA 6 |  | 16 | 0 | 60 |  | -1.476 | 0.000965059 |  | 225 | Right superior frontal gyrus, dorsolateral, BA 6,8 (83)  Right supplementary motor area, BA 6,8 (55) |  | 14/16 |
| Left superior frontal gyrus, medial, BA10 |  | -6 | 50 | 4 |  | -1.816 | 0.000139356 |  | 315 | Left superior frontal gyrus medial, BA10,32(115)  Left anterior cingulate / paracingulate gyri, BA10,32(147) |  | 15/16 |
| Left superior parietal gyrus, BA7 |  | -32 | -66 | 56 |  | -1.719 | 0.000216782 |  | 157 | Left superior parietal gyrus, BA7(123) |  | 15/16 |
| Right amygdala, BA 34 |  | 28 | 0 | -18 |  | -1.222 | 0.003431916 |  | 36 | Right amygdala, BA 34,36,48(30) |  | 15/16 |
| Subgroup meta-analysis of studies without comorbidity | | | | | | | | | | | | |
| FED < HC | | | | | | | | | | | | |
| Left insula, BA 48 |  | -44 | 0 | -4 |  | -2.203 | 0.000005186 |  | 2103 | Left insula, BA 38, 45,47,48(743)  Left rolandic operculum, BA 48 (180)  Left superior temporal gyrus, BA 21, 22,38,48 (275)  Left inferior frontal gyrus, opercular part, BA 6,44,48 (136)  Left inferior frontal gyrus, triangular part, BA38,45,47,48 (96)  Left inferior frontal gyrus, orbital part, BA38,47(84)  Left temporal pole, superior temporal gyrus, BA 21, 38, 48 (279) |  | 13/13 |
| Left parahippocampal gyrus, BA 37 |  | -24 | -40 | -8 |  | -2.249 | ~0 |  | 781 | Left parahippocampal gyrus, BA 20,30,37 (136)  Left hippocampus, BA 20,30,37(101)  Left fusiform gyrus, BA 20,30,37(161)  Left lingual gyrus, BA 30,37(29)  Left cerebellum, hemispheric lobule IV / V, BA 30(14) |  | 13/13 |
| Right parahippocampal gyrus, BA 20 |  | 26 | -30 | -14 |  | -1.894 | 0.000211596 |  | 789 | Right parahippocampal gyrus, BA 20,27,30,35,36,37(138)  Right hippocampus, BA 20,27,30,35 (31)  Right fusiform gyrus, BA 20,30,37(65)  Right cerebellum, hemispheric lobule III, BA 30(36)  Right cerebellum, hemispheric lobule IV / V, BA 30(35)  Right lingual gyrus, BA 27,30(33) |  | 13/13 |
| Right superior frontal gyrus, dorsolateral, BA 6 |  | 18 | 0 | 62 |  | -1.986 | 0.000082552 |  | 240 | Right superior frontal gyrus, dorsolateral, BA 6,8 (129)  Right supplementary motor area, BA 6 (48) |  | 13/13 |
| Subgroup meta-analysis of studies with comorbidity | | | | | | | | | | | | |
| FED < HC | | | | | | | | | | | | |
| Left superior frontal gyrus, medial, BA 10 |  | -6 | 50 | 4 |  | -2.327 | ~0 |  | 987 | Left superior frontal gyrus, medial, BA 10 (283)  Left superior frontal gyrus, medial orbital, BA 10 (128)  Left anterior cingulate / paracingulate gyri, BA 32 (338)  Left gyrus rectus, BA 11 (113)  Left olfactory cortex, BA 25 (87) |  | 6/6 |
| Left superior parietal gyrus, BA 7 |  | -34 | -64 | 54 |  | -2.045 | 0.000036120 |  | 359 | Left superior parietal gyrus, BA 7 (243)  Left inferior parietal gyri, BA 7 (98)  Left angular gyrus, BA 7 (19) |  | 6/6 |
| Right superior frontal gyrus, orbital part, BA 25 |  | 12 | 14 | -22 |  | -2.573 | ~0 |  | 2011 | Right gyrus rectus, BA 11 (235)  Right striatum (142)  Right amygdala, BA 34 (178)  Right superior frontal gyrus, medial orbital, BA 11 (150)  Right superior frontal gyrus, orbital part, BA 11 (73)  Right anterior cingulate / paracingulate gyri, BA 11 (133)  Right olfactory cortex, BA 25 (82)  Right parahippocampal gyrus, BA 28 (67)  Right superior frontal gyrus, medial, BA 10 (39)  Right hippocampus, BA 34 (25)  Right temporal pole, superior temporal gyrus, BA 34 (26)  Right inferior frontal gyrus, orbital part, BA 11 (11) |  | 6/6 |

Abbreviations: FED = first episode depression; HC=healthy control; MNI = Montreal Neurological Institute; SDM = signed differential mapping; BA= Brodmann area; FEW-corrected *p* < 0.05
